# Anasarca, and Lymphadenopathy in a Kidney Transplant Patient: A Diagnostic and Therapeutic Challenge

**DOI:** 10.3389/ti.2022.10148

**Published:** 2022-03-17

**Authors:** Sophie Huegli, David A. Jaques, Sophie De Seigneux, Fadi Haidar

**Affiliations:** ^1^ Division of Nephrology and Hypertension, Department of Medicine, Geneva University Hospitals, Geneva, Switzerland; ^2^ Division of Transplantation, Department of Surgery, Geneva University Hospitals, Geneva, Switzerland

**Keywords:** kidney transplant, immunosuppressants, kaposi sarcoma, anasarca, bloody pleural effusion, axillary lymphadenopathy

## Case Report

A 57-year-old male kidney transplant recipient, originating from Congo and living in Switzerland for 10 years, was referred to our emergency department on the 26th of March 2021 for dyspnea. The clinical examination revealed anasarca progressing over 2 months, bilateral lower limbs edema and hydrocele. There were no skin or mucosal lesions at presentation. Symptoms started shortly after the patient returned from a 2-week trip to Kinshasa, Congo. His past medical history was relevant for a living-donor kidney transplantation in March 2019, in the context of end stage renal disease due to diabetic and hypertensive nephropathy. The patient had a history of subclinical C4d positive acute antibody mediated rejection (ABMR), treated with 1 dose of Rituximab in October 2019. Comorbid conditions included insulin-dependent type 2 diabetes mellitus, hypertension, treated obstructive sleep apnea, and stable monoclonal gammapathy (MGUS) with IgG lambda (62.3 mg/L). Immunosuppression at admission consisted of Ciclosporin, Mycophenolate mofetil, and Prednisone 5 mg/day.

**FIGURE 1:**
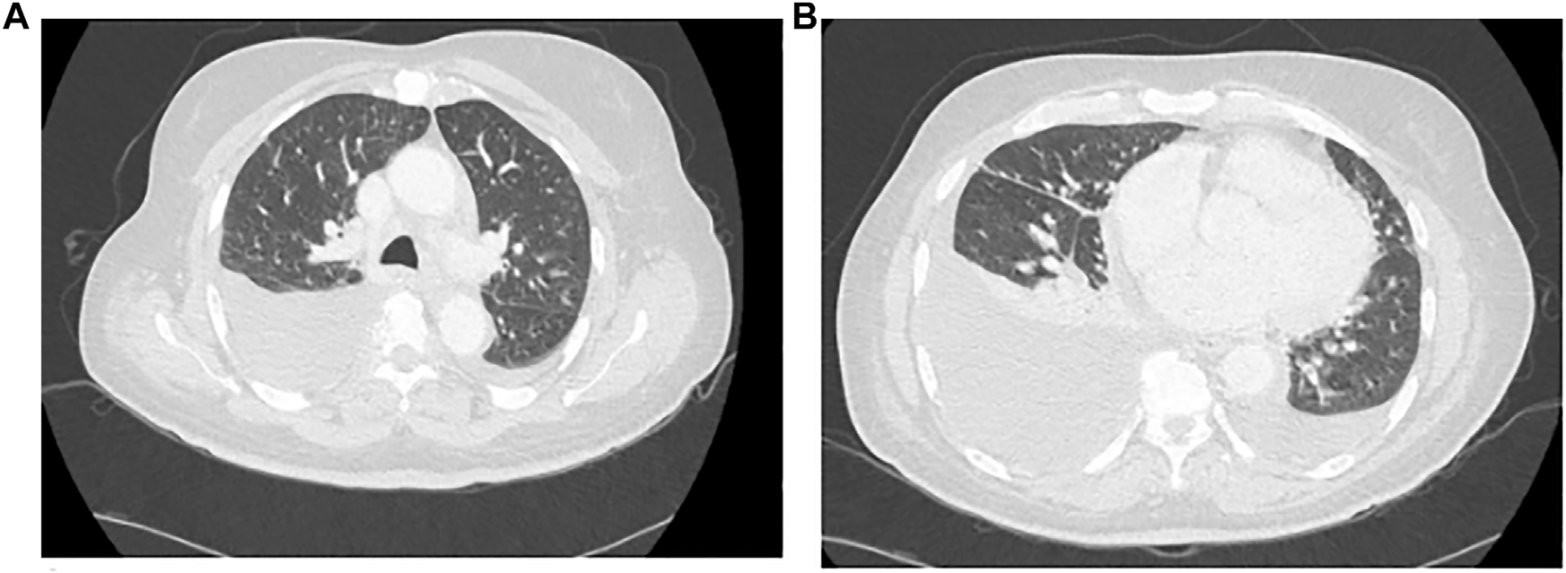
Chest CT Scan, **(A)** Thoracic high, **(B)** Thoracic low.

Diagnostic work-up prior to hospital admission included an ultrasound of the lower limbs excluding thrombosis, normal transthoracic echocardiography as well as blank urinalysis without proteinuria. A CT scan was performed on 10 March 2021 (Figures 1A,B) and showed bilateral pleural effusion, predominantly on the right side with passive contact atelectasis. There were no ground glass opacities.

In the emergency department, initial blood work-up showed normal renal function, with normal electrolytes. Serum albumin was normal. The blood count showed mild thrombocytosis and mild hypochromic microcytic anemia with a Hb of 122 g/L. Leucocyte count was 5.1 g/L, with mild eosinophilia (1.16 g/L), and lymphopenia (0.3 g/L). CRP was mildly elevated at 17 mg/L. EBV and CMV viremias were negative. Quantiferon tuberculosis (TB) test was negative.

A right thoracentesis of 3 L was performed, relieving the dyspnea. Pleural fluid was bloody (1.45 × 10^7/L erythrocytes) and filled criteria for an exudate. Pleural culture, PCR for TB, adenosine deaminase as well as cytology were all negative in the pleural fluid analysis.

Bronchoscopy with broncho-alveolar lavage was obtained and showed a cell count of 10^7/L, with 83% macrophages, 16% lymphocytes, and 1% neutrophiles.

**FIGURE 2:**
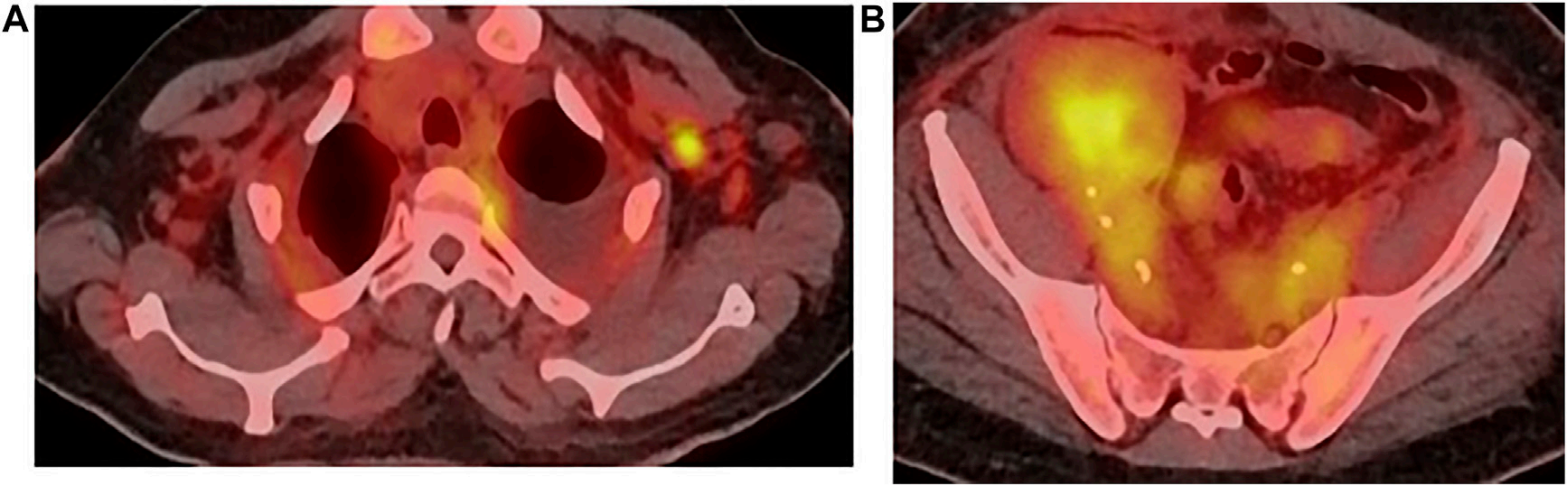
PET-CT, **(A)** axillary, **(B)** inguinal.

A whole-body 18-FDG PET-CT was obtained (Figures 2A,B), and showed pathological diffuse peritoneal hypermetabolism, as well as hypermetabolic right inguinal and left axillary lymph nodes.

## Test Questions

(1) The blood lymphocyte subsets show: CD3^+^ = 1,050/ml; CD4^+^ = 550/ml; CD8^+^ = 500/ml; CD19^+^ = 4/ml; CD56^+^ CD16^+^ (NK cells) = 124/ml. These results are compatible with:
  (a) CD8^+^ cells depletion
  (b) CD4+ cells depletion
  (c) Severe B lymphocyte (CD19+) cells depletion
  (d) Abnormal NK cells level
  (e) Post transplant lymphoproliferative disease (PTLD)
(2) The broncho-alveolar lavage showed a cell count of 10^7/L, with 83% macrophages, 16% lymphocytes, and 1% polyneutrophiles. These results:
  (a) Are compatible with community acquired pneumonia
  (b) Are compatible with Mycobacterium tuberculosis infection
  (c) Are compatible with SARS-CoV-2 infection
  (d) Are compatible with intra-alveolar hemorrhage
  (e) Are normal
(3) What procedure would you recommend as the next step towards diagnosis?
  (a) Kidney graft biopsy
  (b) Left axillary lymph node biopsy
  (c) Abdominal surgical exploratory laparotomy
  (d) Bone marrow biopsy
  (e) Presumptive antituberculous treatment
(4) In terms of diagnosis, which answer is correct in this case?
  (a) Because of his African origin, the patient is at increased risk for Kaposi sarcoma (KS)
  (b) KS is secondary to HPV infection
  (c) PTLD can be excluded because EBV viremia is negative.
  (d) TB is excluded because of the negative TB Quantiferon test
  (e) CMV infection is a possible diagnosis
(5) How would you manage immunosuppression in the case of suspicion of malignancy or disseminated infection?
  (a) Stop Mycophenolate mofetil, keep Prednisone and ciclosporin
  (b) Increase immunosuppression by increasing the ciclosporin trough level
  (c) Stop all immunosuppression
  (d) Increase immunosuppression by doubling the dose of MMF
  (e) Increase immunosuppression by switching from ciclosporin to tacrolimus

## Figures and Tables

**FIGURE 3:**
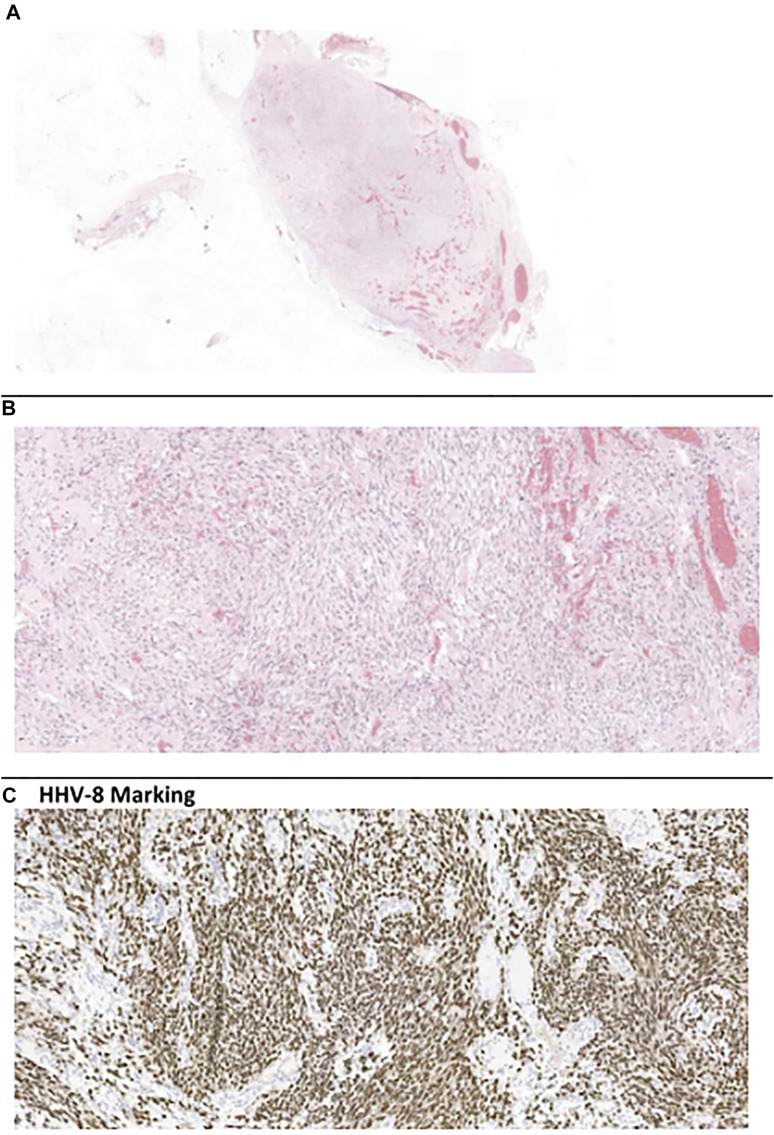
Left axillary lymph node biopsy. **(A, B)** H-E coloration, **(C)** HHV-8 marking.

## Data Availability

The original contributions presented in the study are included in the article, further inquiries can be directed to the corresponding author.

## Appendix

### Answers and Discussion

#### Question 1

**The correct answer is c.**

The blood lymphocyte subsets show severe depletion in CD19^+^ cells (B lymphocytes) with a level below 90/ml. This is secondary to the Rituximab injection that the patient received in October 2019 to treat subclinical ABMR.

PTLD Lymphoma cannot be diagnosed on these blood lymphocyte subsets alone.

#### Question 2

**The correct answer is e.**

The bronchoalveolar lavage (BAL) results are normal. However, the cell count in the BAL is of limited diagnostic value in clinical practice, in particular for infectious etiologies.

BAL cellularity in COVID-19 is predominantly neutrophilic (70%) and to a lesser extent macrophagic (27%) (1). In TB, BAL is characterized by increased neutrophil frequencies and decreased proportions of lymphocytes and macrophages (2).

The results for community acquired pneumonia would also be predominantly neutrophilic. There is no evidence of intra-alveolar hemorrhage, given the absence of red blood cells.

In the bronchoscopy, bacterial culture, PCR for tuberculosis, PCR for *Pneumocystis jirovecii*, and PCR for *Legionella pneumophilia* were all negative. Viral PCRs for HSV-1 and 2, SARS-CoV-2, CMV, adenovirus, and various respiratory viruses were also negative.

#### Question 3

**The correct answer is b.**

Given the results of the PET-CT, the next step towards diagnosis is a lymph node biopsy. We chose the left axillary lymph node as it was readily accessible. The right inguinal lymph node was not clinically observable. This strategy was overall less invasive than abdominal surgical exploratory laparotomy.

Given the normal complete blood count, we would advise against a bone marrow biopsy as a next step.

Presumptive antituberculous treatment could be discussed. In our patient, the results of the pleural effusion and bronchoscopy were negative for TB, and a pleural biopsy showed no sign of TB. Furthermore, this strategy would not rule out other causes of lymphadenopathy and pleural effusion, so a lymph node biopsy was necessary.

As none of the findings indicate renal dysfunction, kidney graft biopsy would be of low yield. In our patient, a left axillary lymph node biopsy was obtained (Figures 3A,B), and showed infiltration of the whole lymph node by fusocellular cells, with strong nuclear staining for HHV-8 (Figure 3C), establishing the diagnosis for KS. HHV-8 viremia was positive (12,000 GEq/ml).

Because of the bloody pleural effusion and the peritoneal hypermetabolism on the PET-CT, we concluded on a KS with visceral involvement. Biopsy of the pleura also showed KS, and so we did not do any gastro-intestinal investigations, but peritoneal KS was suspected.

#### Question 4

**The correct answer is a.**

KS is associated with HHV-8 infection. The endemic form is common in sub-Saharan Africa (3). KS occurring in solid organ transplant recipients is uncommon, but the risk is 100–200 times greater than that of the general population. It is more frequent in developing countries, with rates mirroring the HHV-8 seroprevalence (<5% in North America and Northern Europe, 30% in countries in the Mediterranean and the Middle East, and 50–60% in Sub-Saharan Africa). It usually appears in the first year post-kidney transplant but has been reported up to 18 years after the transplant (3). The classical presentation is cutaneous, with angiomatous lesions predominating on the legs and lymphedema. Visceral disease without cutaneous involvement occurs in about 10% of patients, mostly in the lymph nodes, intestines, and lungs (4).

Our patient had axillary and inguinal lymphadenopathy and anasarca. This situation evokes several differential diagnoses. CMV infection could explain diffuse lymphadenopathy but does not usually cause anasarca. Furthermore, CMV viremia was negative.

PTLD would be a reasonable differential diagnosis. Most cases post-transplant are associated with EBV infection, however, 20–40% are EBV negative. EBV negative cases occur more frequently after the first year of transplantation. In our patient, EBV viremia was negative, but this does not exclude PTLD. The gold standard for diagnosis of PTLD is the lymph node biopsy.

TB is also a probable differential diagnosis, given the bloody pleural effusion, lymphadenopathy, and the African origin of our patient. It cannot be ruled out only by a negative Quantiferon test. In our case, pleural fluid culture and PCR for TB were negative, but these tests have very low sensitivity and cannot exclude a pleural TB. Adenosine deaminase in the pleural fluid, which was also negative in our case, has a high negative predictive value if the effusion is lymphocyte dominant, but would be difficult to interpret in this case, given the bloody pleural effusion. Bronchoscopy and pleural biopsy are warranted to exclude TB. They were negative for TB in our case.

#### Question 5

**The correct answer is a.**

In the case of a suspicion of malignancy or diffuse infection in a kidney transplant recipient, there is a strong indication that to reduce immunosuppression, which we did initially by interrupting mycophenolate mofetil, and keeping ciclosporin with prednisone 10 mg/day. To stop all immunosuppression would not be appropriate, as it would greatly increase the risk of graft loss.

After the diagnosis of KS was made, ciclosporin was switched to everolimus. mTor inhibitors were found to have an anti-tumoral effect on cutaneous KS in a small case study of 15 kidney-transplant recipients (5).

Doxorubicine is considered a first line treatment for KS and is usually associated with a slow response. After a multidisciplinary discussion, chemotherapy was started, with liposomal doxorubicin 20 mg/m^2^ every 2 weeks.

Unfortunately, the patient did not respond well to chemotherapy, with the persistence of lymph node involvement and recurrence of symptomatic pleural effusion. His general condition deteriorated and did not allow for second line treatment. As he deteriorated, the immunosuppression was completely withdrawn, without any rejection episodes. He died approximately 6 months after the KS diagnosis was made of a sudden cardiac arrest after bronchoaspiration.

## References

[1] Dentone C Vena A Loconte M Grillo F Brunetti I Barisione E Bronchoalveolar Lavage Fluid Characteristics and Outcomes of Invasively Mechanically Ventilated Patients with COVID-19 Pneumonia in Genoa, Italy. BMC Infect Dis (2021) 21(1):353. 10.1186/s12879-021-06015-9 33858331 PMC8049078

[2] Seok-Youg E Kong JH Hong MS Lee YJ Kim JH Hwang SH Neutrophils Are the Predominant Infected Phagocytic Cells in the Airways of Patients with Active Pulmonary TB. Chest (2010) 137(1):122–8. 10.1378/chest.09-0903 19749004 PMC2803122

[3] Etta E Alayande DP Mavhandu-Ramarumo LG Gachara G Bessong PO HHV-8 Seroprevalence and Genotype Distribution in Africa, 1998-2017: A Systematic Review. Viruses (2018) 10(9):458. 10.3390/v10090458 PMC6164965 30150604

[4] Sheperd FA Maher E Cardella C Cole E Greig P Wade JA Treatment of Kaposi's Sarcoma after Solid Organ Transplantation. J Clin Oncol (1997) 15(No 6):2371–7. (June). 10.1200/JCO.1997.15.6.2371 9196152

[5] Stallone G SchenaA InfanteB Di PaoloS LoverreA MaggioG Sirolimus for Kaposi’s Sarcoma in Renal-Transplant Recipients. N Engl J Med (2005) 352:1317–23. 10.1056/nejmoa042831 15800227

